# Guide Cells Support Muscle Regeneration and Affect Neuro-Muscular Junction Organization

**DOI:** 10.3390/ijms22041939

**Published:** 2021-02-16

**Authors:** Flavio L. Ronzoni, Nefele Giarratana, Stefania Crippa, Mattia Quattrocelli, Marco Cassano, Gabriele Ceccarelli, Laura Benedetti, Jens Van Herck, Maria G. Cusella De Angelis, Marco Vitale, Daniela Galli, Maurilio Sampaolesi

**Affiliations:** 1 Human Anatomy Unit, Department of Public Health, Experimental and Forensic Medicine, University of Pavia, 27100 Pavia, Italy; flavio.ronzoni@unipv.it (F.L.R.); gabriele.ceccarelli@unipv.it (G.C.); laura.benedetti@unipv.it (L.B.); cusella@unipv.it (M.G.C.D.A.); 2 Department of Biomedical Sciences, Humanitas University, 20090 Milan, Italy; 3 Translational Cardiomyology laboratory, Department of Development and Regeneration, Stem Cell Institute, KULeuven, 3000 Leuven, Belgium; nefele.giarratana@kuleuven.be; 4 Centro Dino Ferrari, Stem Cell Laboratory, Unit of Neurology, Department of Pathophysiology and Transplantation, University of Milan, Fondazione IRCCS Ca’ Granda Ospedale Maggiore Policlinico, 20122 Milan, Italy; 5 Unit of Pathogenesis and therapy of primary immunodeficiencies, San Raffaele Telethon Institute for Gene Therapy, Vita Salute San Raffaele University, 20133 Milan, Italy; crippa.stefania@hsr.it; 6 Cincinnati Children’s Hospital Medical Center, Department of Pediatrics, Heart Institute, University of Cincinnati College of Medicine and Molecular Cardiovascular Biology Division, Cincinnati, OH 45229, USA; Mattia.Quattrocelli@cchmc.org; 7 Department of Biosciences, University of Milan, 20133 Milan, Italy; marco.cassano@unimi.it; 8 Laboratory of Reproductive Genomics, Department of Human Genetics, KU Leuven, 3000 Leuven, Belgium; jens.vanherck@kuleuven.be; 9 Department of Medicine and Surgery, Division of Anatomy, University of Parma, 43100 Parma, Italy; marco.vitale@unipr.it

**Keywords:** guide cells, neuro-muscular junctions, mesoangioblasts, muscle injury, scRNA-seq

## Abstract

Muscular regeneration is a complex biological process that occurs during acute injury and chronic degeneration, implicating several cell types. One of the earliest events of muscle regeneration is the inflammatory response, followed by the activation and differentiation of muscle progenitor cells. However, the process of novel neuromuscular junction formation during muscle regeneration is still largely unexplored. Here, we identify by single-cell RNA sequencing and isolate a subset of vessel-associated cells able to improve myogenic differentiation. We termed them ‘guide’ cells because of their remarkable ability to improve myogenesis without fusing with the newly formed fibers. In vitro, these cells showed a marked mobility and ability to contact the forming myotubes. We found that these cells are characterized by CD44 and CD34 surface markers and the expression of Ng2 and Ncam2. In addition, in a murine model of acute muscle injury and regeneration, injection of guide cells correlated with increased numbers of newly formed neuromuscular junctions. Thus, we propose that guide cells modulate de novo generation of neuromuscular junctions in regenerating myofibers. Further studies are necessary to investigate the origin of those cells and the extent to which they are required for terminal specification of regenerating myofibers.

## 1. Introduction

Unraveling the regenerative processes in skeletal and cardiac muscles represents an intriguing and ambitious frontier. In order to define novel therapeutic strategies, one fundamental step is to understand the properties of plasticity in post-natal progenitor cells.

Recently, many post-natal stem cells (i.e., bone marrow derived stem cells, mesoangioblasts, adipose stem cells, cardiac stem cells, and skeletal myoblasts) were used in several clinical trials to treat cardiac and skeletal muscle diseases [1,2,3,4,5,6,7]. Bone marrow- and adipose-derived stem cells showed an efficient outcome in differentiating towards different muscle commitments. However, skeletal muscle progenitors are less prone to differentiate into different lineages, probably due to their strong differentiation commitment and the existence of cardiac progenitor cells is still debated [8,9,10,11]. Therefore, lately, it is still unclear whether a fate switch between skeletal and cardiac adult myogenesis is possible [12,13]. However, it has been recently demonstrated that some neck muscles (trapezius and sternocleidomastoid) derive from progenitors of the pharyngeal mesoderm. In particular, the trapezius muscle group is clonally related to myocardium of the venous pole of the heart [14]. Thus, the cell population that gives rise to trapezius and sternocleidomastoid represents a group of common skeletal and cardiac muscle progenitors. Furthermore, different exosome and circulating miRNA molecular mechanisms have been proposed as crucial players of progenitor cell commitments [15]. In this regard, we recently described a method to generate bipotent cardiac/skeletal muscle progenitors from murine- and human-induced pluripotent stem cells [16]. Although more studies are needed, this work opens new frontiers for the use of mesodermal origin cells in muscular regeneration. Furthermore, the regeneration of cardiac and skeletal muscles requires correct innervation. To date, pure pacemaker and neuro-muscular junction (NMJ) progenitor populations have not been identified, due to the paucity of the cells and the complexity of the model system to reconstruct.

In 2013, using the embryoid-bodies differentiation system, [17] a cardiac pacemaker progenitor population was isolated from embryonic stem cells (ESCs). Although differentiation potential of stem cells towards a nodal or pacemaker type has been extensively characterized [18], the clinical applicability of this approach is still limited. Interestingly, ESCs and induced pluripotent stem cells can form functional NMJs in skeletal muscle both in vitro and in vivo [19]. However, since these cells could lead to teratoma formation, their applicability slows down in long-term therapies. Notably, it has been demonstrated that both skeletal muscle and cardiac progenitors can adopt neural characteristics both in vitro and in vivo, reinforcing the idea that adult muscle stem cells can change their commitment. In particular, Lavasani et al. in 2014 [20] showed that human muscle-derived stem/progenitor cells (hMDSPCs) acquire neuronal and glial characteristics in vitro, promote axonal regeneration, and reduce muscle atrophy once transplanted in a murine denervated gastrocnemius. More recently, Kelder et al. in 2015 [21] proposed that the myocardial cells of the sinus venosus participate to the nodal extension of the atrio-ventricular node highlighting the dual differentiation potential of those cells.

Among muscle stem cell types, mesoangioblasts (MABs) —vessel-associated stem cells that can be isolated from the dorsal aorta during development or from post-natal cardiac and skeletal muscles [13,22]—present the major advantage of infiltrating a target tissue, thanks to their physiological association to vessels. Indeed, MABs have been systemically delivered in animal models of limb girdle muscular dystrophy and they efficiently restored alpha-sarcoglycan protein levels [23]. Moreover, in a dog model of Duchenne muscular dystrophy, MAB injections in the femoral artery determined a functional amelioration of the disease [24]. In addition, murine-heart-isolated MABs showed sino-atrial like phenotype in vitro [25] suggesting that cardiac MABs present neural properties.

What drives the plasticity properties of the post-natal progenitor cells and what is their role in muscle regeneration needs to be unraveled. Hence, in this study, we focused on uncommitted progenitors among the heterogeneous muscle stem cell pool characterized by single cell RNAseq analysis. We tested the in vivo effect of a subset of vessel-associated cells on murine acute regenerating skeletal muscles upon CTX-induced injury. Here we show for the first time that this cell population expressing CD44, CD34, Sox2, and Ncam2, named guide cells, is able to improve myogenic differentiation of C_2_C_12_ cells without directly fusing with the newly formed myotubes. In particular, guide cells are characterized by high motility allowing the interaction with all the surrounding cells when injected in an acute model of muscular regeneration, they increase the number of NMJs, and they contribute to muscle hypertrophy.

## 2. Results

### 2.1. Isolation of Mesoangioblast Guide Cells

**Figure 1:**
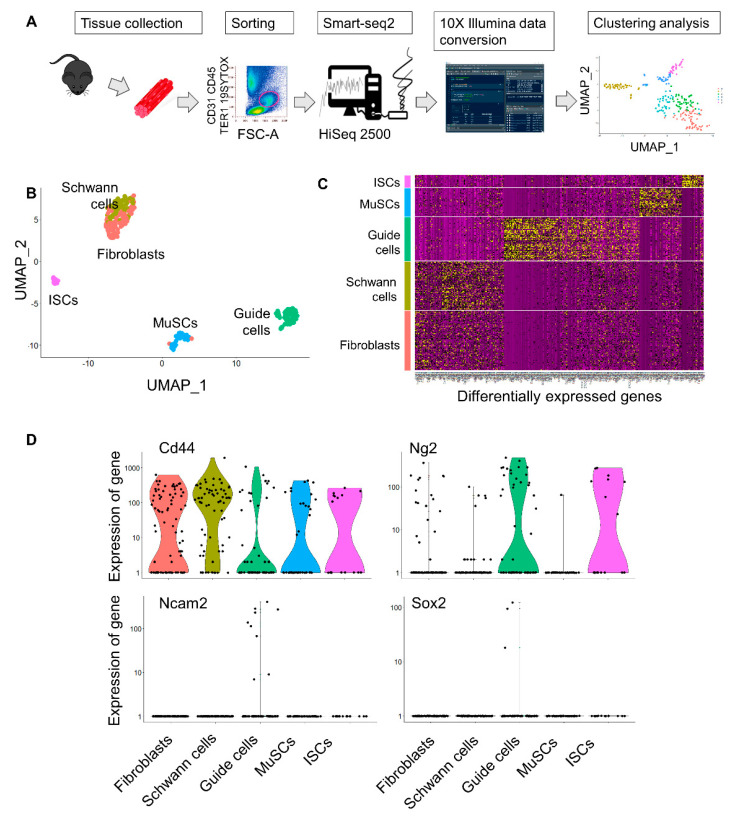
Single-Cell RNA Sequencing reveals the presence of guide cells expressing CD44, Ng2, and Ncam2. (**A**) Flowchart showing the isolation of single cells from murine hind limbs with Smart-seq2 yielding cellular and phenotypic relationships. (**B**) UMAP plot and k-means clustering of 256 cells from murine skeletal muscle identifying five clusters, fibroblasts, Schwann cells, guide cells, activated satellite cells (MuSCs), and interstitial stromal cells (ISCs, as reported in [26]). Every point represents one cell. (**C**) Heatmap of k-means clusters of differentially expressed marker genes. (**D**) Violin plot with median visualizing marker genes (Cd44, Ng2, Ncam2, and Sox2) for the identified clusters.

**Figure 2:**
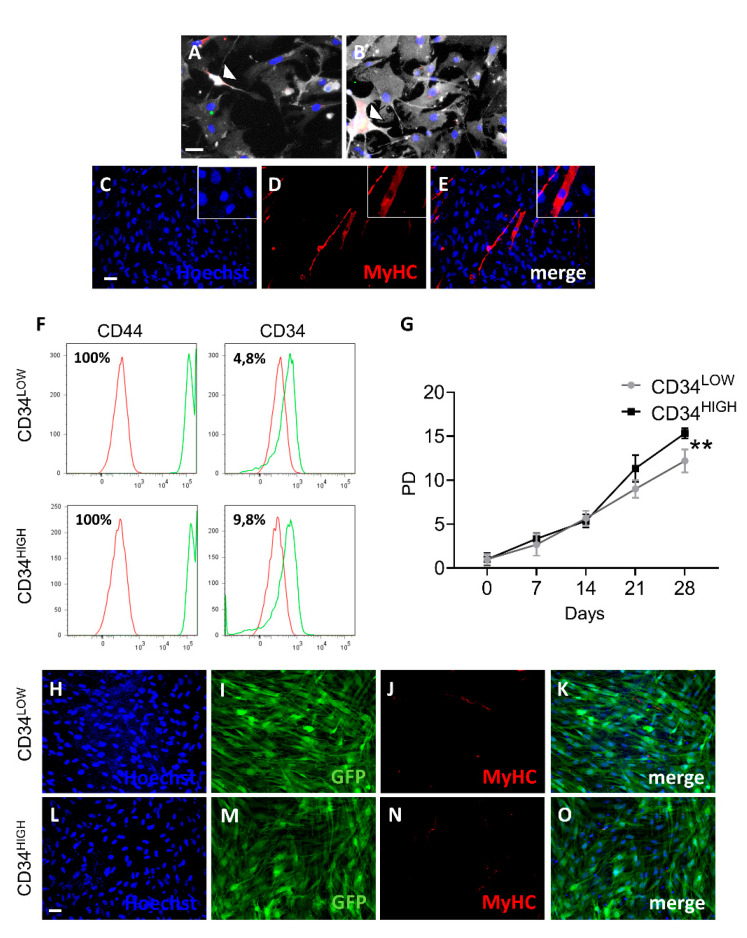
Characterization of the CD34LOW and CD34HIGH positive guide cells. (**A,B**) Confocal images of cardiac (panel A) and skeletal muscle (panel B) mesoangioblasts showing co-expression of CD44 (green), CD34 (far-red), and Ncam2 (red). 5-azacytidine induced differentiation of the cardiac mesoangioblasts. Arrowheads indicate triple-positive cells. Scale bar (40 µm) is the same for panels A and B. (**C**–**E**) 5-azacytidine induced differentiation of the cardiac mesoangioblasts. (**C**) Hoechst nuclei staining, (**D**) myosin heavy chain (MyHC) immunofluorescence, (E) merge of panels C-D. Scale bar (40 μm in C) is the same for panels D and E. (**F**) Fluorescence-activated cell sorting (FACS) analysis of the surface markers (CD44 and CD34) in the guide cells. Green peaks indicate the positive cells for CD34 or CD44 with respect to isotype negative control in red. (**G**) Population doublings of the CD34LOW and CD34HIGH cells; ** *p* ≤ 0.01. (**H**–**O**) Myosin expression analysis in the CD34LOW and CD34HIGH positive cells. (H and L) Hoechst nuclei staining of CD34LOW (H) and CD34HIGH cells (L); (I and M) GFP immunofluorescence of the CD34LOW (**I**) and CD34HIGH cells (M); (**J** and **N**) myosin heavy chain (MyHC) immunofluorescence analysis of the CD34LOW (**J**) and CD34HIGH cells (**N**) after 5-azacytidine treatment; (**K**) merge of H, I, and J panels; (**O**) merge of L, M, and N panels; scale bar in L (20 μm) is the same for all the panels.

**Figure 3:**
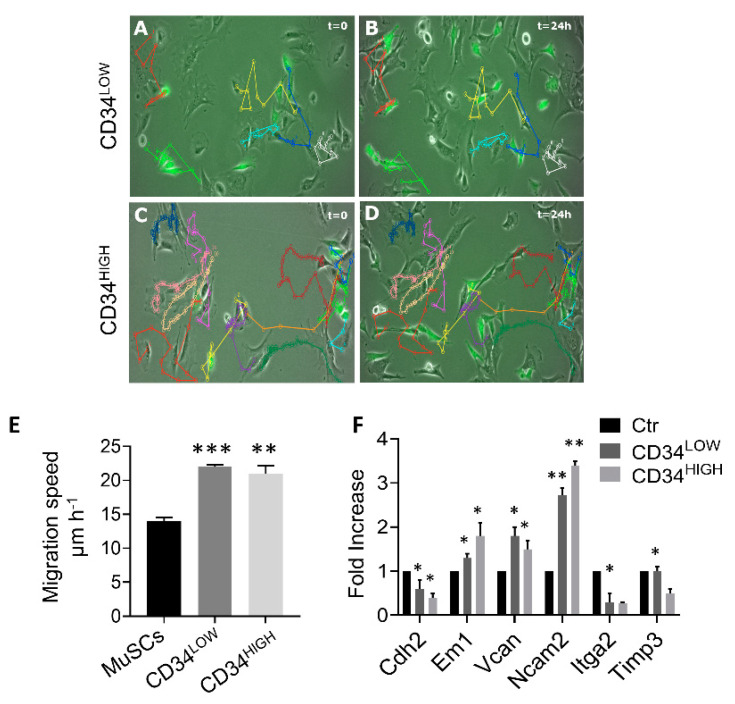
In vitro migration ability of CD34LOW and CD34HIGH positive cells. (**A**–**D**) Pictures representative of the movie analysis. Color pathways report the cell movements (each color indicates a different cell) after 24 h. Colored pathways are reported on the same microscopic field both at *t* = 0 (panels A and C, respectively, for CD34LOW and CD34HIGH cells) and *t* = 24 h (panels B and D, respectively, for CD34LOW and CD34HIGH cells). (**E**) Quantitative analysis of the guide cell migration speed (expressed as µm h-1) and of the MuSCs (indicated with a black bar in **E**) ** *p* ≤ 0.01; *** *p* ≤ 0.001. (**F**) qRT-PCR analysis for extracellular matrix and adhesion molecule genes in cardiac mesoangioblasts (ctrl, black bar), CD34LOW (dark grey bar), and CD34HIGH (light grey bar) guide cells. N-cadherin (Cdh2), Emilin1 (Em1), versican (Vcan), neural cell adhesion molecule (Ncam2), integrin alpha 2 (Itga2), metalloproteinase inhibitor (Timp3). * *p* < 0.05; ** *p* ≤ 0.01.

We recently reported single cell RNAseq analysis in dystrophic and healthy murine skeletal muscles [26] and we reanalyzed the cell types based on the differentially expressed genes using UMAP (uniform manifold approximation and projection) dimension reduction, as reported in the flowchart (Figure 1A). Clustering resulted in five groups that were annotated as fibroblasts, Schwann cells, activated satellite cells (MuSCs), interstitial stromal cells (ISCs), or the novel guide cells, characterized by a distinct transcriptome profile (Figure 1B,C). Guide cells cluster was characterized by the expression of several marker genes including Cd44, Ng2, Ncam2, and Sox2 (Figure 1D). In order to investigate the physiological role of this cell population we isolated interstitial muscle cells as previously described [18,19,20]. In particular, muscles were collected from 1-week-old C57 mice and, under a stereo-microscope, dissociated into small fragments and plated on 1% gelatin [27]. After initial growth of fibroblasts, poorly adherent cells appeared and were isolated by gently pipetting. With a time-lapse system we could observe that, although scanty, there were spindle cells characterized by high-speed movements that seemed to direct and “guide” the behavior of the other cells (Video S1). Interestingly, these muscle cell populations’ rare spindle cells showed co-expression of CD44, Sox2, and Ncam2 (Figure 2A,B) as identified in the guide cells cluster by scRNAseq analysis. CD44 is one of the most important markers for myoblast cell motility. In fact, CD44-/- muscle stem cells show defects in cell movement as well as in vitro differentiation. Sox2 and Ncam2 are considered neural stem cell markers [28,29]. However, those interstitial cells expressed terminal differentiation markers (i.e., myosin heavy chain) (Figure 2C–E) when exposed to low serum and 5-azacytidine treatment suggesting a contamination of muscle mesoangioblasts. Thus, to further isolate “guide” cells we proceeded to differentiate the cells with two rounds of culture in 5-azacytidine and low serum medium for 5 days. Undifferentiated cells were then detached by trypsin treatment and cloned by progressive dilution. Two cell populations were derived from five mice, then selected and characterized (Figure 2, panels F–O). Both cell populations were 100% positive for CD44 but differently expressed CD34 (4.8% in the case of CD34LOW cells and 9.8% in the case ofCD34HIGH cells) (Figure 2F). Instead both cell populations were negative for CD13, CD31, and CD45 (data not shown). Figure 2G reports the growth curves of the two cell populations. The trends were constant and similar until seven population doublings (PD). After that point, the proliferation rate became slightly divergent for the two guide cell populations, and particularly slower for the CD34HIGH cells. Once exposed to low serum medium both CD34LOW and CD34HIGH cells were viable but did not express myosin anymore (Figure 2, panels H and L). Interestingly, both CD34LOW and CD34HIGH cell populations maintained highspeed movements (Video S2 and Video S3). A quantitative analysis of the cells’ speed is reported in Figure 3. In particular, the speed of the CD34LOW (GFP-labeled, Figure 3A,B) and CD34HIGH cells (GFP-labeled Figure 3C,D) was measured together with the speed of the MuSCs. Both CD34LOW andCD34HIGH cells showed significantly higher speed compared to MuSCs (Figure 3E). Moreover, we investigated the expression of extracellular matrix and adhesion molecules involved in cell migration by PCR arrays and by quantitative RT-PCR (qRT-PCR). Results are shown in Figure 3F. The expression of genes involved in cell adhesion and migration has been analyzed—N-cadherin (Cdh2) is involved in neural cell adhesion and migration [30]. Emilin-1 (Em1) regulates smooth muscle cell activity in vessel diameter determination [31], the proteoglycan versican (Vcan) is a crucial marker involved in heart development [32], neural cell adhesion molecule 2 (NCAM2) [33] was the highest expressed marker in both cell populations, and integrin alpha2 (Itga2) and metalloproteinase3 inhibitor (Timp3) regulate endothelial cell migration [34].

### 2.2. Guide Cells Increase Fusion Index of C_2_C_12_ Myoblasts and H4ven Mesoangioblasts

**Figure 4:**
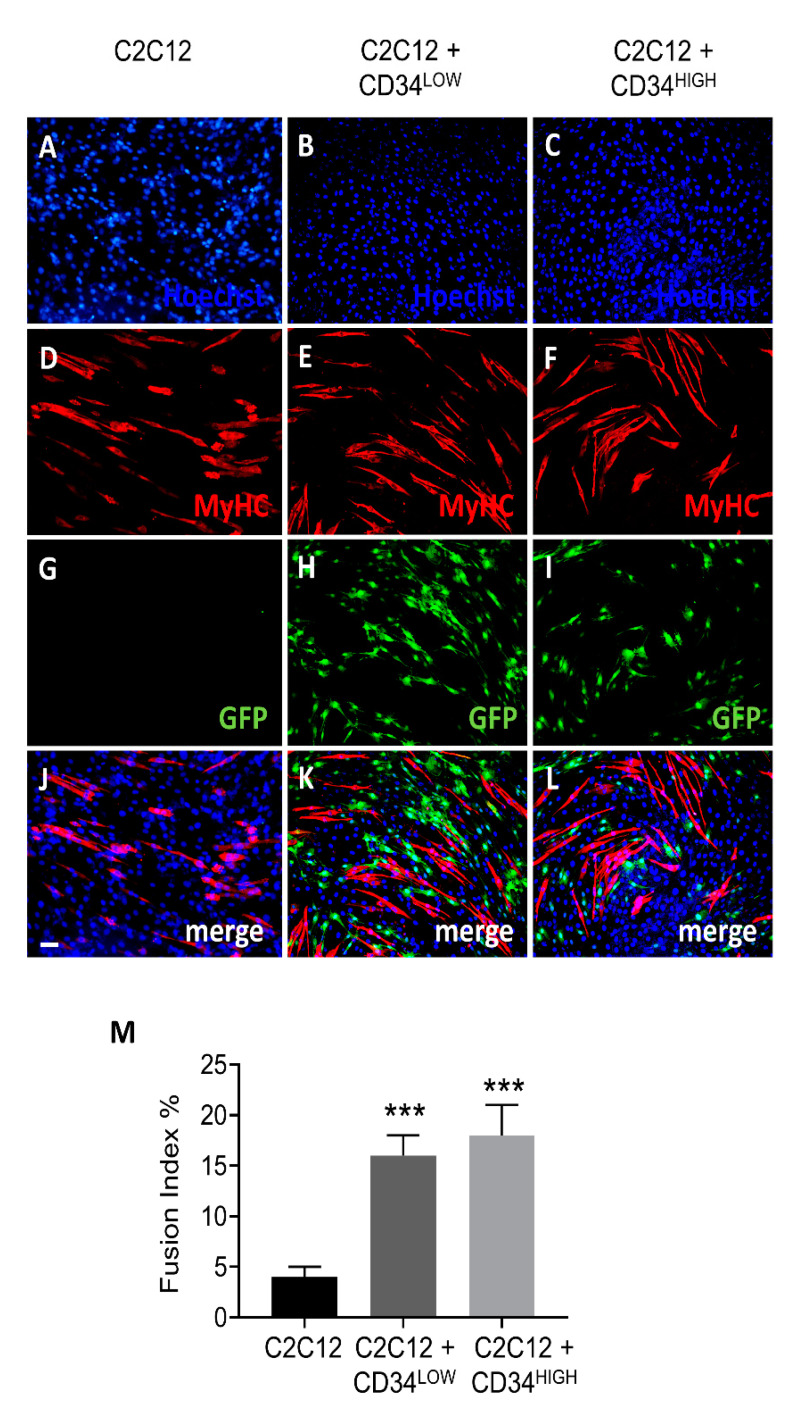
Myogenic differentiation (5 days) of C2C12 cells co-cultured with guide cells. (**A**–**C**) Hoechst nuclei staining of C2C12 (**A**), C2C12 co-cultured with CD34LOW (**B**), or with CD34HIGH cells (**C**); (**D**–**F**) myosin heavy chain (MyHC) immunofluorescence analysis on C2C12 (D), on C2C12 co-cultured with myosin heavy chain (MyHC) immunofluorescence analysis on C2C12 (**D**), on C2C12 co-cultured with CD34LOW (E), or with CD34HIGH cells (**F**); (**G**–**I**) GFP immunofluorescence analysis of C2C12 (**G**), C2C12 co-cultured with CD34LOW (**H**), or with CD34HIGH (**I**) guide cells; (**J**) merge of panels A, D, and G. (**K**) merge of panels B, E, and H. (**L**) merge of panels C, F, and I. (**M**) Fusion index of C2C12, C2C12 co-cultured with CD34LOW or with CD34HIGH cells; *** *p* < 0.001. Scale bar 30 μm for all panels.

To verify if the guide cells could orchestrate cell differentiation, we tested this capability by co-culture CD34LOW and CD34HIGH cells with C_2_C_12_ and H4ven mesoangioblasts. C_2_C_12_ cells represent a good model of skeletal muscle differentiation, in fact they can be maintained in a proliferative state or induced to differentiate (myotubes formation) by serum deprivation. On the other side, H4ven cells represent *β*-sarcoglycan (Sgcb)-null cardiac progenitors that display an anomalous activation of skeletal muscle genes that are commonly silenced in healthy cardiac progenitors. This cardiac mesoangioblast cell line efficiently differentiates into skeletal muscle in vitro and. This is due to the lack of miR669q and the down-regulation of miR669a [35]. Both C_2_C_12_ and H4ven cells formed myosin positive myotubes (Figure 4 and Figure S1A,D,J). CD34LOW and CD34HIGH cells (labelled in green with a GFP lentiviral vector [36] did not express myosin or fuse with C_2_C_12_ or H4ven, in fact we could not observe green or orange myotubes (Figure 4 and Figure S1H–L). However, the presence of both guide cell populations determined a significant increase in the number of myotubes formed by H4ven and C_2_C_12_ (Figure 4 and Figure S1E,F). A quantitative analysis of the myoblast fusion index is reported in Figure 4G and Figure S1G. The fusion index was calculated as the percentage of nuclei in MyHC-positive myotubes applying the following formula: (nuclei within MF20- stained myotubes/total number of nuclei) × 100.

### 2.3. Guide CD34HIGH Cells Increased α-BTX Positive Junctions In Vivo

Since the guide cells increased myoblast and mesoangioblast differentiation in vitro, we injected them in cardiotoxin-injured tibialis muscles of nude mice to see if CD34LOW and CD34HIGH cells were able to increase muscle tissue regeneration in vivo. As control, we injected PBS in the contra-lateral cardiotoxin-injured muscles. In particular, the muscle injected with CD34HIGH cells was 30% longer and 50% larger compared to the control. When immunostained for GFP, muscles injected both with CD34LOW and CD34HIGH cells showed GFP-positive cells (Figure S2A–C,E–G). However, we could not detect any GFP-positive fibers, suggesting that the hypertrophic effect on muscle mass could not be due to neo-formed GFP fibers. This again suggested to us that the cells could exert an indirect effect on tibialis fibers. Hematoxylin/eosin staining (Figure S2D,H) showed morphology of the muscles.

**Figure 5:**
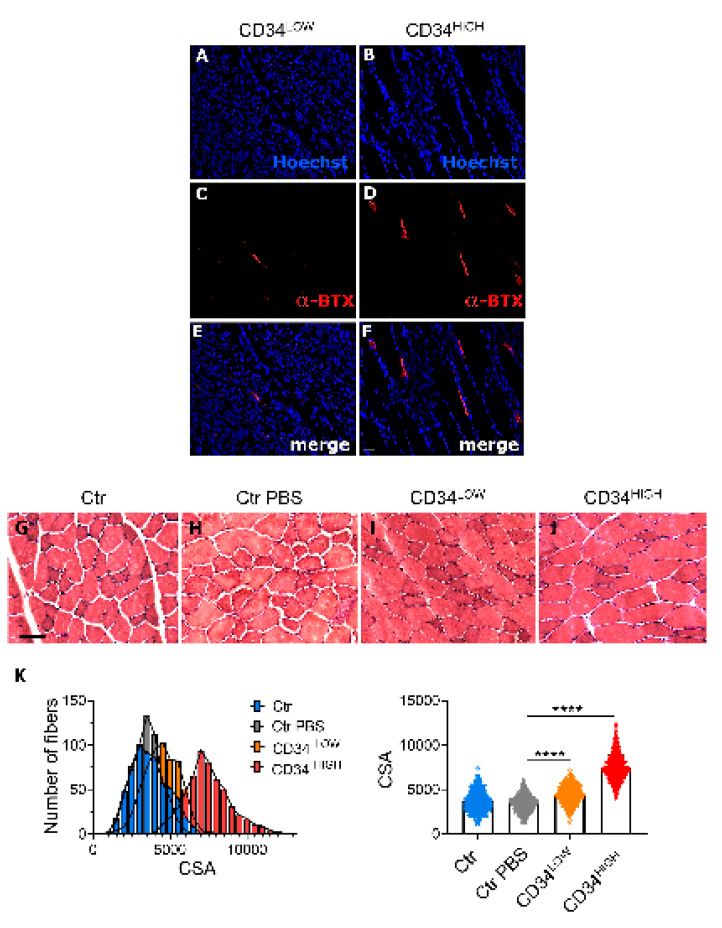
Neuro-muscular junction localization in muscles injected with CD34^LOW^ and CD34^HIGH^ cells. (**A**,**B**) Hoechst nuclei staining of longitudinal sections of muscles injected with CD34^LOW^ (A) and CD34^HIGH^ cells (B); (**C**,**D**) bungarotoxin staining of the same sections as in A and B; (**E**) merge of A and C; (**F**) merge of B and D. Scale bar (30 μm in F) is the same for all the panels. (**G**–**J**) Examples of hematoxylin/eosin staining of *TA* muscles after cardiotoxin (CTX) treatment and injected with PBS (H), with CD34^LOW^ cells (I) CD34^HIGH^ cells (**J**) or uninjected (**G**). (**K**) Morphometric analysis of muscles shown in G–J. In the left histogram the distribution of cross sectional area (CSA) values of un-injected (Ctr, blue bars), PBS-injected (Ctr PBS, grey bars), CD34^LOW^- cells-injected mice (CD34^LOW^, orange bars) and CD34^HIGH^-cells injected mice (CD34^HIGH^, red bars) was determined on H&E-stained *Tibialis Anterior (TA)* muscle bundles of cross cryosections from six mice per group. In the right panel, the average of CSA values is represented; *N* = 600 per group, * *p* < 0.05; ** *p* < 0.01; **** *p* < 0.0001.

Muscle weight can be increased with 6 weeks of contractile activity stimulation of the sciatic nerve [37,38]. Since only the muscle injected with CD34HIGH cells showed hypertrophy without the presence of GFP fibers, we envisaged that the effect of CD34HIGH cells could be exerted at the level of neuro-muscular junctions. Notably, CD34HIGH cells expressed Cx40 after serum deprivation while CD34LOW cells did not. We thus investigated the number of neuro-muscular junctions in the tibialis muscles injected both with CD34LOW and CD34HIGH guide cells (Figure 5). The acetylcholine receptor is one of the synapse markers and it is bound by alpha-bungarotoxin [37]. We observed a 30% increase in density of α-bungarotoxin positive structures in tibialis injected with the CD34HIGH cells, confirming our hypothesis. Interestingly, we observed a significantly bigger cross sectional area (CSA) of muscle fibers in CTX-treated tibialis anterior (TA) of mice injected with CD34HIGH cells compared to TA that were either not injected, or injected with PBS or with CD34LOW cells (Figure 5G–K and Figure S2I–K).

### 2.4. Guide Cells Differently Express Neural Markers

**Figure 6:**
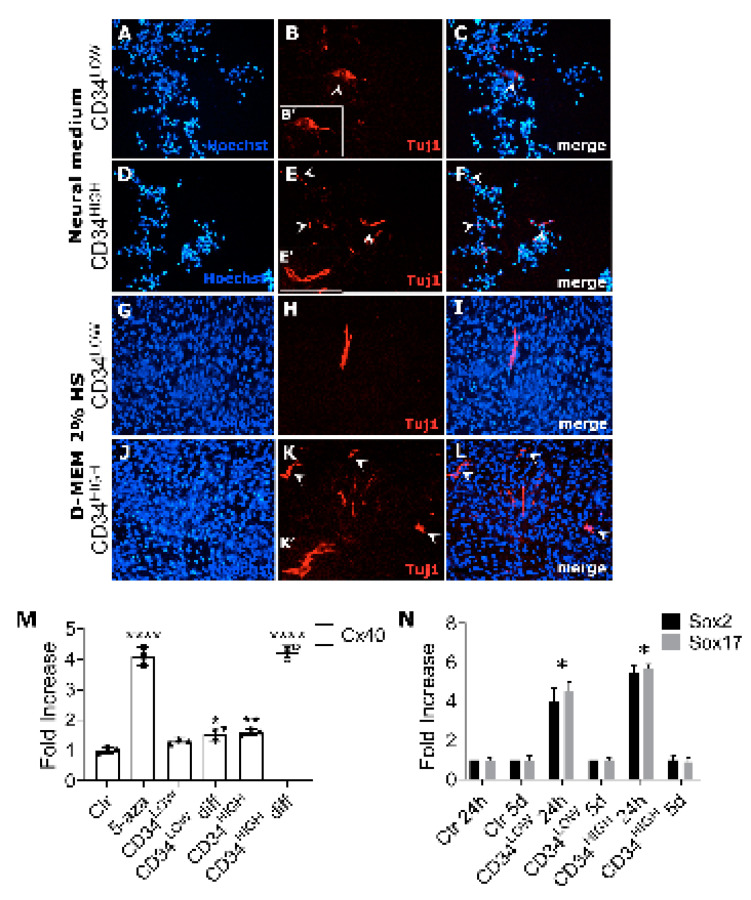
Expression of neural markers in guide cells under different culture conditions. (**A**,**D**,**G**,**J**), Hoechst nuclei staining of CD34LOW (**A**,**G**) and CD34HIGH cells (**D**,**J**) cultured in neural medium (**A**,**D**) or D-MEM 2% Horse Serum (**G,J**); (**B**,**E**,**H**,**K**) anti-Tuj1 immunofluorescence of CD34LOW (**B**,**H**), and CD34HIGH cells (**E**,**K**) cultured in neural medium (**B**,**E**) or D-MEM 2% horse serum (**H**,**K**). Insets B’, E’, K’ represent sub-regions of panels B, E, K respectively; (**C**) merge of A and B panels; (**F**) merge of D and E panels; (**I**) merge of panels G and H; (**L**) merge of panels J and K. Scale bar (30 μm), in L, is the same for all the panels. (**M**) Connexin40 mRNA levels in the original population used as reference (**C**), after 5-azacytidine treatment (5-aza) and in the guide cells used in the experiments, grown both in proliferating medium and in low serum medium (CD34LOW diff and CD34HIGH diff cells). (**N**) Relative expression of Sox2 (white bar) and Sox17 (black bar) in cardiac mesoangioblasts (ctrl) or guide cells cultured for 24 hours (24h) in proliferating medium and 5 days in low serum medium (5 d); * *p* < 0.05, ** *p* < 0.01; **** *p* < 0.0001.

Since guide cells determined a significant increase of CSA (Figure 5) and TA weights (Figure S2) of injected muscles without participating directly to myogenesis, we wondered if the observed muscle hypertrophy could be due to nervous stimulation following cell injection. We then studied neural differentiation in vitro of the two guide cell populations, characterizing the expression of specific markers after treatment with neural inductive medium or low serum medium. Both guide cell populations were positive for III beta-tubulin (Tuj1) either after neural induction medium addition or serum deprivation (Figure 6A-L) confirming that CD34LOW and CD34HIGH cells could show a neural phenotype in appropriate culture conditions. Moreover, we observed that CD34HIGH cells showed up-regulation of the cardiac pacemaker marker Connexin 40 [39] when induced to differentiate by low serum treatment (Figure 6M). Finally, since *NCAM2* expression was increased in the guide cells, we also investigated the expression of the neural stem cell markers like Sox2 and Sox17 (Figure 6N). Sox2 is involved in the maintenance and differentiation of neural stem cells [29]. Sox17 is involved in oligodendrocytes survival in models of de-myelination and multiple sclerosis. Both the genes were significantly up-regulated in the proliferating guide cells (24 h). After 5 days of differentiation for both CD34LOW and CD34HIGH cells, the levels decreased and became similar to the control.

## 3. Discussion

Although there is a wide debate on the role of cell therapy for cardiac and muscle disease, many points still need to be clarified. In particular, embryonic stem cells and reprogrammed fibroblasts would represent the best tool for therapy if their capacity to form teratoma could be abolished. On the other side, the use of adult progenitors is limited by the reduced plasticity of the cells, although the exposure to recombinant proteins can improve their myogenic differentiation potential [39,40,41,42,43]. So far, data on the injection of adult satellite stem cells and the influence of new neuro-muscular junction formations are limited. Also, there are no evidence of the participation of cardiac stem cells in the regeneration of the heart conduction system. Notwithstanding this fact, recently, it has been shown that trapezius and sternocleidomastoid muscles have common progenitors with the venous pole of the heart [14]. Interestingly, it has been demonstrated that, both in vitro and in chicken embryos, mesoderm head progenitors contribute to the heart and head muscle formation [44]. This suggests that there is a common precursor for heart and muscular tissues, opening new scenarios on stem-cell plasticity.

Here we studied a sub-population proposed as guide cells, present both in skeletal and cardiac muscles that disclose “leading” capabilities and neural properties in vitro. In particular, our results show that these progenitors are able to improve skeletal muscle differentiation of C_2_C_12_ and H4ven cardiac mesoangioblasts [35]. Moreover, guide cells had their own characteristic high-speed movements and positively affected the muscle mass when injected in CTX-treated muscles. Cell movement is not only crucial in muscle regeneration but also in many other biological processes including cancer invasive progression [45] and wound healing/regeneration processes [46].

We should also consider the difference between migration (directional movement) and random motion (motility) that are both connected to the cell speed. In fact, different speed motilities characterize specific cell types. For example, neutrophils, that are the fastest moving leukocytes, display a speed range between 900–1200 mm/h [40]. Notably, mouse neonatal neuronal cells show an average speed at 48–68 μm/h. Our study shows that CD34^LOW^ and CD34^HIGH^ guide cells move much slower at 20–22 μm/h and their speed is in the range of human mesenchymal stem cell motility reported at 4–37 μm/h [47]. Nevertheless, guide cells move faster than primary satellite cells and direct them to a more efficient differentiation. The fact that CD34LOW and CD34HIGH guide cells are 100% positive for CD44, a compelling marker for cell migration [48], reinforces the idea that these cells have motility features. This feature could be directly involved in skeletal muscle hypertrophy and the increase of neural muscular junction formations observed when guide cells were transplanted in regenerating muscles.

Unfortunately, the literature regarding the nervous stimulation that induces skeletal muscle hypertrophy, is scarce. However, it has been demonstrated that pharmacologic stimulation of *β*2-adrenoceptor (*β*2-AR) induces skeletal muscle hypertrophy in different mammalian species suggesting a possible association between hypertrophy and nervous system stimulation [49]. In the future, it will be interesting to specify which type of fibers cause the guide-cells-mediated muscle hypertrophy and determine the order of the absolute force of treated muscles. This will unveil the functional effects of guide cell administration and the involved molecular mechanisms.

Further studies will be necessary to investigate the origin of these cells and their involvement in tissue specification. Ultimately, these cells have been isolated even from the heart and since they express Cx40 and other neural markers upon myogenic induction, they could eventually participate to the cardiac conduction system formation. We consequently plan to investigate the guide cells’ function in the sinus node. To date, the literature on the progenitors for heart impulse specification in vivo is still lacking, making it difficult to set up experiments. Hopefully, in the near future, when the proteasome of guide cells is fully defined and gain and loss of function experiments performed, it will be possible to deepen their role in the cardiac conduction system formation.

## 4. Materials and Methods

### 4.1. Single Cell RNAseq Analysis

Single cell RNAseq analysis was performed as recently reported [50]. Briefly, LIN- muscle cells were single-cell sorted by FACS in 96-well plates (4titude). Each well contained 4 mL lysis buffer (0.4% Triton X-100 in RNase-free water supplemented with 10 mM biotinylated Oligo-dT (IDT), 10 mM dNTPs (Thermo-Fisher Scientific, Geel, Belgium) and 0.5 U/mL RNase inhibitor (Takara, Shiga, Japan). cDNA libraries were generated based on the SMART-seq2 protocol [26]. Analysis of SmartSeq2 scRNA-seq data was performed with the Seurat [51] R package (version 3.0.1). The raw counts previously gathered [26] were made compatible (ENSEMBL# to gene Symbol, https://www.biotools.fr/mouse/ensembl_symbol_converter) for importing data into Seurat. Expression value scaling and normalization, Principal component-A (PCA) and Uniform Manifold Approximation and Projection (UMAP) dimensionality reductions and clustering were performed. After filtering cells containing a high content of mitochondrial genes and a high content of ERCCs, the remaining cells were further analyzed. The expression values were renormalized, rescaled, and re-clustered and cells were manually annotated based on their differentially expressed genes.

### 4.2. Cytofluorimetric Analysis

Cytofluorimetric analysis has been performed as previously described [52] with some modifications. Briefly, guide cells were detached with PBS/5 mM Ethylenediaminetetraacetic acid (EDTA) and resuspended in 0.5% Bovin Serum Albumin (BSA) in Phosphate-buffered saline (PBS) (FACS buffer). 0.3 × 106 cells were incubated with FITC-monoclonal antibodies for the following CD antigens: CD44 (BD Biosciences, San Jose, CA, USA) and CD34 (BD Biosciences, San Jose, CA, USA) for 30 min at 4 °C. Analysis was performed on a FACScan (BD Biosciences, San Jose, CA, USA).

### 4.3. Cell Cultures

Cells were isolated as previously described [23,53]. Muscles isolated from one-week-old mice, were rinsed in PBS and sharply dissected into 1–2-mm-diameter pieces with a scalpel. Fragments containing small vessels were transferred to a Petri dish coated with 1% gelatin in the presence of 20% FBS-DMEM (Dulbecco’s Modified Eagle Medium) (Sigma, Milan, Italy) plus 5 mM glutamine and antibiotics. These muscle fragments were cultured for 8 days and after the initial outgrowth of fibroblast-like cells, small round and refractive cells appeared. This cell population was easily collected by gently pipetting of the original culture. Cells were induced to differentiate with 10 μM 5′-azacytidine (Sigma A2385, Milan, Italy) for 5 days. 5′-azacytidine is a DNA hypomethylating agent capable of inducing or enhancing cardiac differentiation by regulating bone morphogenic protein (BMP) signaling molecules and specific cardiac marker genes. Undifferentiated cells were cloned by progressive dilution. For co-culture experiments, guide cells (100% green fluorescent protein (GFP) labelled with LV-CMV-GFP vector) were seeded in the same 30-mm dish with C_2_C_12_ or H4ven mesoangioblasts in a ratio 1:4 and cultured in DMEM (Sigma, Milan, Italy), 2% horse serum (Euroclone, Milan, Italy) 100 U/mL penicillin streptomycin, and 200 mM L-glutamine for 5 days. Neural differentiation was induced by culturing the cells in Dulbecco’s modified Eagle’s medium/F12 (Sigma, Milan, Italy) with 2% FBS (Euroclone, Milan, Italy) or in DMEM with 2% FBS (Euroclone, Milan, Italy), 100 U/mL penicillin streptomycin, and 200 mM L-glutamine [54].

### 4.4. Cells and Tissue Immunofluorescence

Cell immunofluorescence was performed as previously described [55] with some modifications. Fixed cells were permeabilized with 1% BSA and 0.2% TritonX-100 in PBS for 5 min and then blocked in 10% donkey serum for 1 h. After 1.5 h of incubation with primary antibodies CD44 (BD Biosciences, San Jose, CA, USA), CD34 (Thermo-Fisher Scientific, Geel, Belgium), Ncam2 (Abcam, ab204446. Cambridge, UK), MF20 mouse monoclonal antibody for Myosin Heavy Chain DSHB cat#MF-20; RRID AB_2147781), Tuj1 (Sigma, Milan, Italy), diluted in 1% donkey serum, samples were washed three times with PBS and incubated with anti-mouse or anti-rabbit secondary antibody (1:1000) conjugated with alexa fluor 488 (Thermo-Fisher Scientific, Geel, Belgium) or alexa fluor 594 (Thermo-Fisher Scientific, Geel, Belgium) fluorochromes. Nuclei were counterstained with Hoechst 33,258 (Sigma, Milan, Italy) 1µg/mL diluted in PBS for 5′ and subsequently and sections were visualized with a Nikon (Milan, Italy) fluorescence microscope. To set up microscope parameters (gain and exposition time) samples without primary antibody incubation were used. Acetylcholine receptors were stained with alpha-bungarotoxin conjugated with alexa fluor 594 (Thermo-Fisher Scientific, Geel, Belgium). Briefly, frozen sections of muscles (TA) were fixed with 2% paraformaldehyde in PBS for 5′, rinsed in PBS, then incubated for 1 h at 4 °C with a 1 μg/mL alpha-bungarotoxin (Thermo-Fisher Scientific, Geel, Belgium) diluted 1:500 in PBS. Sections were rinsed with PBS, nuclei were counterstained with Hoechst (Sigma, Milan, Italy) and slides were observed under a fluorescence microscope (Nikon, Milan, Italy).

### 4.5. Gene Expression Analysis

Total RNA was extracted using RNeasy mini kit (Qiagen, Antwerp, Belgium) according to the manufacturer’s protocol. qRT-PCR reactions were performed in triplicates, using glyceraldehyde-3-phosphate dehydrogenase (GAPDH) as housekeeping gene in a mini-Opticon Instrument (Bio-Rad, Temse, Belgium) with 200 nM of each primer. Results were analyzed using MJ Opticon Monitor Analysis software Version 3.1 (Bio-Rad). Primer sequences were: Sox2 fw: 5′-TGCTGCCTCTTTAAGACTAGGG-3′ Sox2 rev: 5′-TCGGGCTCCAAACTTCTC-3′; Sox17 fw: 5′-CTTTATGGTGTGGGCCAAAG-3′ Sox17 rev: 5′-TTGTAGTTGGGGTGGTCCTG-3′ Cx40 fw: 5′-AGCAGCCAGAGCCTGAAGAA-3′ Cx40 rev: 5′- CAGGACAGTGAGCCAGACCT-3′. Primers were synthesized by PRIMM, Milan, Italy. Extra-cellular matrix (ECM), adhesion and ECM gene expression was analyzed by RT^2^ Profiler™ PCR Arrays from Sabioscience (Qiagen, Antwerp, Belgium) following manufacturer’s instructions.

### 4.6. Motility Assay

Motility assay through time-lapse acquisitions allowed the observation and investigation of fast (minutes) and slow (hours) cellular motility events. Video tracking involved measuring the position of whole cells at a specific time point. Movies were acquired by time-lapse confocal microscopy (Nikon BioStation IM-Q). Frames were taken every 30 min for 3 d (30 min/6 frames/s). The analysis was performed using ImageJ software (NIH, Bethesda, MD, http://www.nih.gov/ij) and the plugin MTrackJ that allows manual tracking of individual cell track as previously described [56]. Analyses were performed on at least six single cells/group on each microscope field and at least three microscope fields were studied for 24 h for each guide cell population. The full length of the track was determined as the distance from the first point to the last point of the track, and the cell speed was measured as µm*hour-1.

### 4.7. In Vivo Experiments

For in vivo experiments 500,000 CD34LOW or CD34HIGH cells were dispensed in PBS and injected in tibialis anterior (TA) muscles of 18 cardiotoxin (CTX, 10 uM)-injured and 6 control (CTR) nude mice. Contra-lateral muscles were used as control. Mice were euthanized by lethal CO_2_ exposure 4 weeks after injection and TA muscles were dissected, then embedded in OCT (Bio Optica, Milan, Italy) and immediately frozen in isopentane cooled in liquid nitrogen and stored at −80 °C for further analysis. The muscles were subsequently cryosectioned at 10 μm in thickness. Serial frozen sections were withdrawn and hereafter fixed with 4% paraformaldehyde following the antibody instruction’s manuals. Immunofluorescence and hematoxylin/eosin staining were performed following our standard protocols [57], and morphometric analysis was carried out analyzing 600 TA sections per group.

Mouse experiments were performed according to international ethical guidelines (EEC Council Directive 86/609; NIH Guide for the Care and Use of Laboratory Animals, 1985). The authorization for animal experimentation was obtained from the Italian Ministry of Health (authorization n°668/2018-PR prot. 5CEED.72).

### 4.8. Statistics

Each experiment was repeated three times. Mean values were compared by ANOVA test to verify the significance of the results. When two groups were compared, a non-parametric *t*-test was performed. The data are indicated as the mean ± SD. All statistical tests were performed with Excel (Microsoft office 2011) and GraphPad Prism 8.0 (GraphPad Software, San Diego, CA, USA).

## Data Availability

Single cell raw data (GEO: GSE147883) are available at the following link: https://www.ncbi.nlm.nih.gov/geo/query/acc.cgi?acc=GSE147883.

## Supplementary Materials

The following are available online at https://www.mdpi.com/1422-0067/22/4/1939/s1, Figure S1: Myogenic differentiation (5 days) of H4ven mesoangioblasts co-cultured with guide cells. Figure S2: Cardiotoxin-injured tibialis anterior muscles injected with guide cells. Video S1: Time-lapse confocal microscopy of CD44, Sox2, and Ncam2 positive cells. Video S2: Time-lapse confocal microscopy of CD44, Sox2, Ncam2 and CD34^LOW^ positive cells. Video S3: Time-lapse confocal microscopy of CD44, Sox2, Ncam2 and CD34^HIGH^ positive cells.
