# Supplementary material for: Guide Cells Support Muscle Regeneration and Affect Neuro-Muscular Junction Organization
Source: Int J Mol Sci. 2021 Feb 16;22(4):1939. doi: 10.3390/ijms22041939 (PMC7920023; doi:10.3390/ijms22041939)
Supplement: Supplementary file 1 [file ijms-22-01939-s001.zip › ijms-1056849-supplementary-for conversion/ijms-1056849-supplementary-for conversion.pdf]

Guide Cells Support Muscle Regeneration and Affect Neuro-Muscular Junction Organization

Flavio L. Ronzoni [1,2,†], Nefele Giarratana [3,4,†], Stefania Crippa [5], Mattia Quattrocelli [6], Marco Cassano [7], Gabriele Ceccarelli [1], Laura Benedetti [1], Jens Van Herck [8], Maria Gabriella Cusella De Angelis [1], Marco Vitale [9], Daniela Galli[9,*] and Maurilio Sampaolesi [1,3,*]

[1] Department of Public Health, Experimental and Forensic Medicine, Human Anatomy Unit, University of Pavia, 27100, Pavia, Italy
[2] Department of Biomedical Sciences, Humanitas University, 20090, Milan, Italy.
[3] Translational Cardiomyology laboratory, Department of Development and Regeneration, Stem Cell Institute, KULeuven, 3000, Leuven, Belgium.
[4] Stem Cell Laboratory, Department of Pathophysiology and Transplantation, University of Milan, Unit of Neurology, Fondazione IRCCS Ca' Granda Ospedale Maggiore Policlinico, Centro Dino Ferrari, 20122, Milan, Italy.
[5] San Raffaele Telethon Institute for Gene Therapy, Unit of Pathogenesis and therapy of primary immunodeficiencies - Vita Salute San Raffaele University, 20133, Milan, Italy.
[6] Department of Pediatrics, University of Cincinnati College of Medicine and Molecular Cardiovascular Biology Division, Heart Institute, Cincinnati Children's Hospital Medical Center, Cincinnati, 45229, OH, USA.
[7] Department of Biosciences, University of Milan, 20133, Milan, Italy.
[8] Department of Human Genetics, Laboratory of Reproductive Genomics, KU Leuven, 3000, Leuven, Belgium.
[9] Department of Medicine and Surgery, Division of Biomedical, Biotechnological and Translational Sciences, University of Parma, 43121, Parma, Italy
† These authors contributed equally to this work
* Corresponding author daniela.galli@unipr.it (D.G.); maurilio.sampaolesi@kuleuven.be (M.S.)

Supplementary Figure Legends

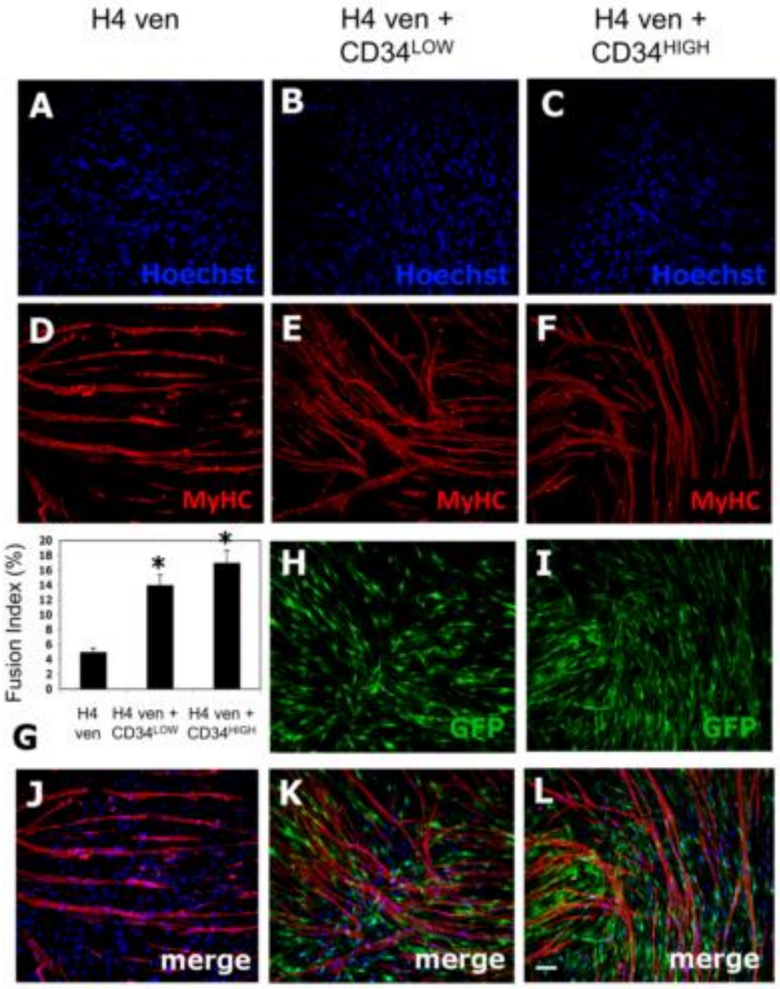

Figure S1: Myogenic differentiation (5 days) of H4ven mesoangioblasts co-cultured with guide cells. (A-C), Hoechst nuclei staining of H4ven (A), H4ven co-cultured with CD34^{LOW} (B), or with and CD34^{HIGH} cells (C). (D-F), Myosin Heavy Chain (MyHC) immunofluorescence analysis on H4ven (D), H4ven co-cultured with CD34^{LOW} (E) or CD34^{HIGH} cells (F). (G), fusion index (see results) of H4ven, H4ven co-cultured with CD34^{LOW} or CD34^{HIGH} cells; *$p<0.03$. (H, I), GFP immunofluorescence analysis on H4ven co-cultured with CD34^{LOW} (H) or CD34^{HIGH} cells (I). (J) merge of A and D panels. (K) merge of B, E and H panels. (L) merge of panels C, F, I. Scale bar = 40 μm.

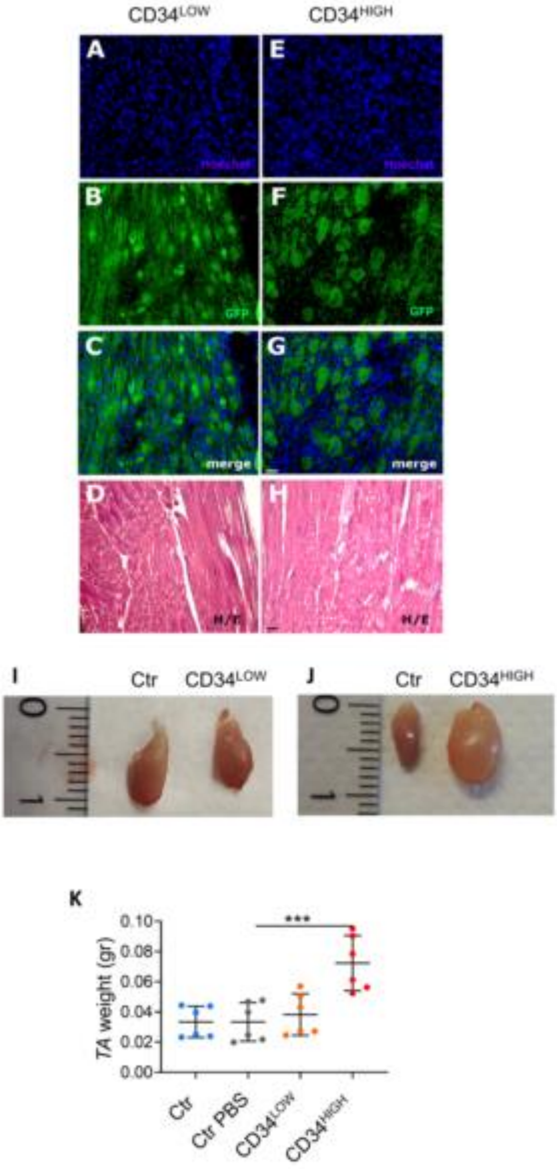

Figure S2: Cardiotoxin-injured *tibialis anterior* muscles injected with guide cells. (A, E), Hoechst staining of muscles injected with CD34^{LOW} (A) and CD34^{HIGH} cells (E). (B, F), GFP immunofluorescence in muscles injected with CD34^{LOW} (B) and CD34^{HIGH} cells (F). (C), merge of A, B panels; (G) merge of E, F panels. Scale bar in G for panels A-G, 50 μm. (D, H), hematoxylin and eosin (H/E) staining of muscles injected with CD34^{LOW} (D) and CD34^{HIGH} cells (H). Scale bar for panels D-H, 100 μm. (I, J) The images show CTX injured *tibialis anterior* muscles, 4-weeks after the injection of PBS (Ctr) or CD34^{LOW} cells (CD34^{LOW}) and CD34^{HIGH} cells (CD34^{HIGH}), respectively. (K) Weight (gr) of TA in un-injected mice (Ctr, blue dots), PBS injected mice (Ctr PBS, grey dots), CD34^{LOW} cells injected mice (CD34^{LOW}, orange dots) and CD34^{HIGH} cells injected mice (CD34^{HIGH}, red dots); N=6 per group, ***$p<0.001$.

Supplementary Video Legends

Video S1: Time-lapse confocal microscopy of CD44, Sox2, and Ncam2 positive cells.

Video S2: Time-lapse confocal microscopy of CD44, Sox2, Ncam2 and CD34^{LOW} positive cells.

Video S3: Time-lapse confocal microscopy of CD44, Sox2, Ncam2 and CD34^{HIGH} positive cells.
